# The effect of human placental chorionic villi derived mesenchymal stem cell on triple-negative breast cancer hallmarks

**DOI:** 10.1371/journal.pone.0207593

**Published:** 2018-11-20

**Authors:** Alaa T. Alshareeda, Emad Rakha, Ayidah Alghwainem, Bahauddeen Alrfaei, Batla Alsowayan, Abdullah Albugami, Abdullah M. Alsubayyil, Mohmed Abomraee, Nur Khatijah Mohd Zin

**Affiliations:** 1 Stem Cell and Regenerative Medicine Department, King Abdullah International Medical Research Centre, King Abdulaziz Medical City, Ministry of National Guard Health Affairs, Saudi Arabia; 2 University of Nottingham and Nottingham University Hospitals NHS Trust, City Hospital, Department of Cellular Pathology, UK, Nottingham, United Kingdom; 3 School of Advance Science and Engineering, Waseda University, Tokyo, Japan; University of South Alabama Mitchell Cancer Institute, UNITED STATES

## Abstract

Mesenchymal stem cells (MSCs) can influence the tumour microenvironment (TEM) and play a major role in tumourigenesis. Triple-negative [Ostrogen receptor (ER-), Progesterone receptor (PgR-), and HER2/neu receptor (HER2-)] breast cancer (TNBC) is an aggressive class of BC characterized by poor prognosis and lacks the benefit of routinely available targeted therapies. This study aims to investigate the effect of human placental chorionic villi derived MSCs (CVMSCs) on the behavior of TNBC *in vitro*. This was done by assaying different cancer hallmarks including proliferation, migration and angiogenesis. Cell proliferation rate of TNBC cell line (MDA-MB231) was monitored in real time using the xCELLigence system. Whereas, Boyden chamber migration assay was used to measure MDA-MB231 motility and invasiveness toward CVMSCs. Finally, a three-dimensional (3D) model using a co-culture system of CVMSCs with MDA-MB231 with or without the addition of human umbilical vein endothelial cells (HUVECs) was created to assess tumour angiogenesis *in vitro*. CVMSCs were able to significantly reduce the proliferative and migratory capacity of MDA-MB231 cells. Co-culturing of MDA-MB231 with CVMSCs, not only inhibited the tube formation ability of HUVECs but also reduced the expression of the BC characteristic cytokines; IL-10, IL-12, CXCL9 and CXCL10 of CVMSCs. These results support the hypothesis that CVMSCs can influence the behavior of TNBC cells and provides a basic for a potential therapeutic approach in a pre-clinical settings. The data from this study also highlight the complexity of the *in vitro* cancer angiogenesis model settings and regulations.

## Introduction

MSCs are non-haematopoetic progenitor cells with the ability to differentiate into a variety of mesenchymal lineage such as, but not limited to, adipocytes, osteocytes, chondrocytes and myocytes **[1, 2]**. Several studies have demonstrated that MSCs isolation could be procured from a variety of adult tissues including bone marrow, liver, dental pulp, adipose-tissue and muscle **[3–9]**. MSCs were also reported to have the ability to trans-differentiate into hepatocyte, neural and astrocytes-like cells *in-vitro* **[10]**. MSCs influence TME by affecting the way cancer cells behave and spread.

Cancer cell proliferation, tumour angiogenesis and invasiveness are prompted by the abundance of growth factors, chemokines, cytokines, metabolites and structural protein components that are released by MSCs within the TME **[11]**. For example, it was reported that MSCs produce tenascin Cs **[12]** which has been associated with breast cancer (BC) metastasis to the lungs **[13]**.

Triple-negative breast cancer (TNBC) is defined by the lack of Ostrogen receptor (ER-), Progesterone receptor (PgR-), and HER2/neu receptor (HER2-). Representing about 15–20% of all BCs, TNBC is regarded as aggressive tumors that are resistant to existing target treatments [14]; TNBC patients have a poorer outcome and relapse fairly quickly while on chemotherapy [15]. Characteristically, TNBC is defined as highly proliferative with a high mitotic count and central necrosis [16], and is associated with a higher degree of angiogenesis than non-TNBC tumors [17].

Angiogenesis plays an important role in tumour growth, since blood vasculature formation is essential in maintaining the influx of essential nutrients to a tumour [18]. Studies have reported the correlation between tumour growth and angiogenesis [19] however; most of the angiogenesis *in vitro* studies carried out only targeted cell differentiation, endothelial cells migration and proliferation. Therefore, a study with a tumor angiogenesis *in vivo* model that includes supporting stromal cells and endothelial cells in addition to tumor cells and the extracellular matrix (ECM) would offer valuable novel prospective.

The current study aims to investigate the effect of human placental derived MSC on the behavior of TNBC cells *in vitro* utilizing different cancer hallmarks including proliferation, migration and angiogenesis. In this study the placental MSCs are isolated from the chorionic villi (CVMSCs), which are located on the fetal side of maternal-fetal interface and human umbilical vein endothelial cells. This sub-type of MSCs was chosen due to the fact that the effect of placental MSCs, Including CVMSCs, on cancers is not well investigated. In addition, the placenta is a very practical source of MSCs, as it is readily available as a discarded medical waste, and a large number of MSCs can be easily isolated from a single placenta. Thus, if CMSCs were shown to attenuate the hallmarks of cancer, their use in a pre-clinical and clinical setting will be very feasible.

## Results

### Cell isolation and characterization

Primary CVMSCs and HUVECs were successfully isolated as described in the methodology section.

#### Characterization of CVMSCs by flow cytometric analysis

**Fig 1:**
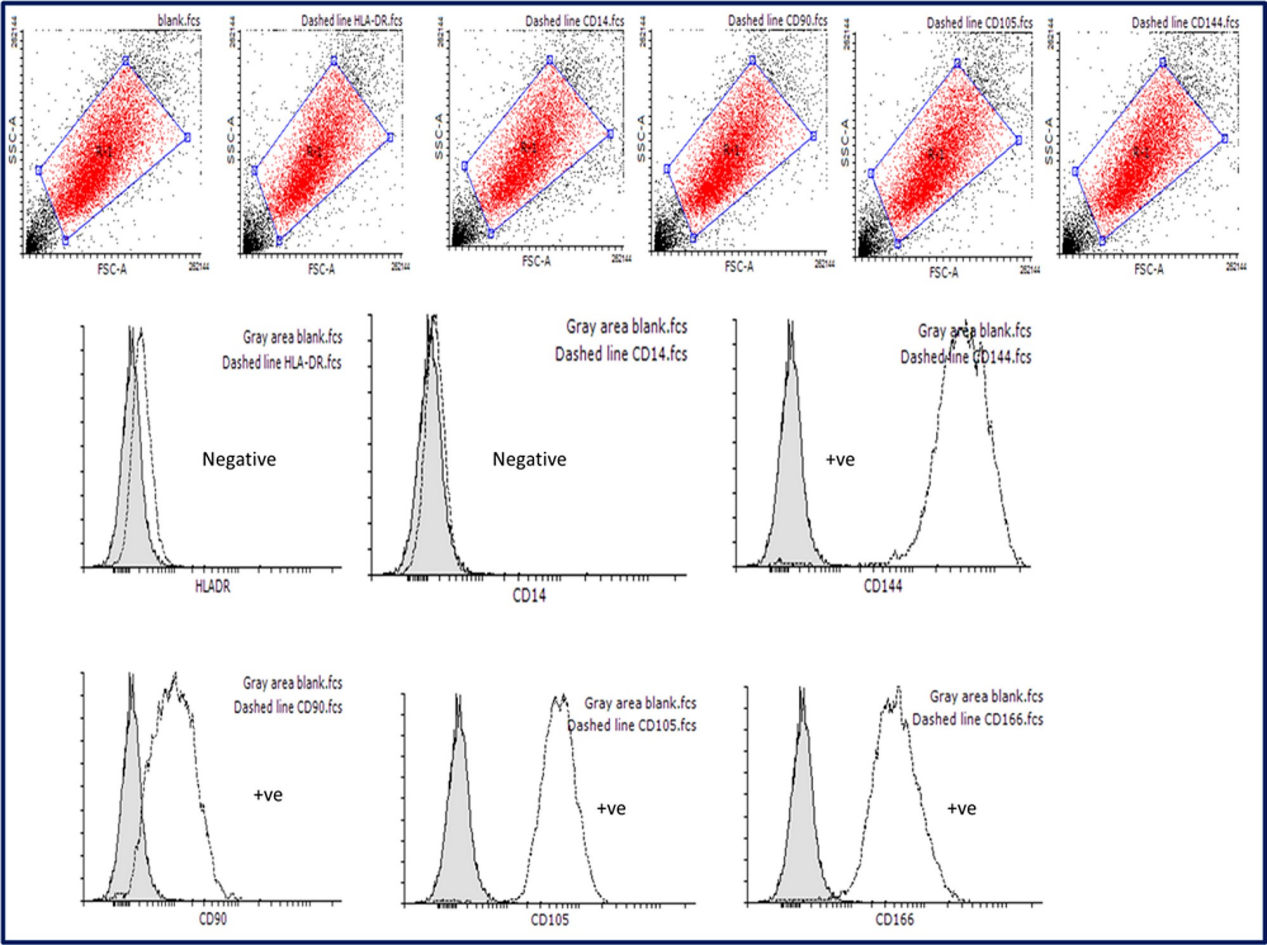
Validation of MSCs markers by flow cytometry. MSCs found positive for the following markers CD144, CD90, CD105, and CD166. In addition, MSCs were negative for HLA-DR, and CD-14 markers. These results are typical for MSCs validation assay.

Successful isolation of MSCs was confirmed with flow cytometric analysis of surface markers. The isolated CVMSCs were positive for the positive markers CD90, CD144, CD105, and CD166 [20] "Fig 1 ", and negative for the negative markers HLA-DR and CD14 [21] "Fig 1 ".

#### Differentiation of human placental chorionic villi derived MSCs

**Fig 2:**
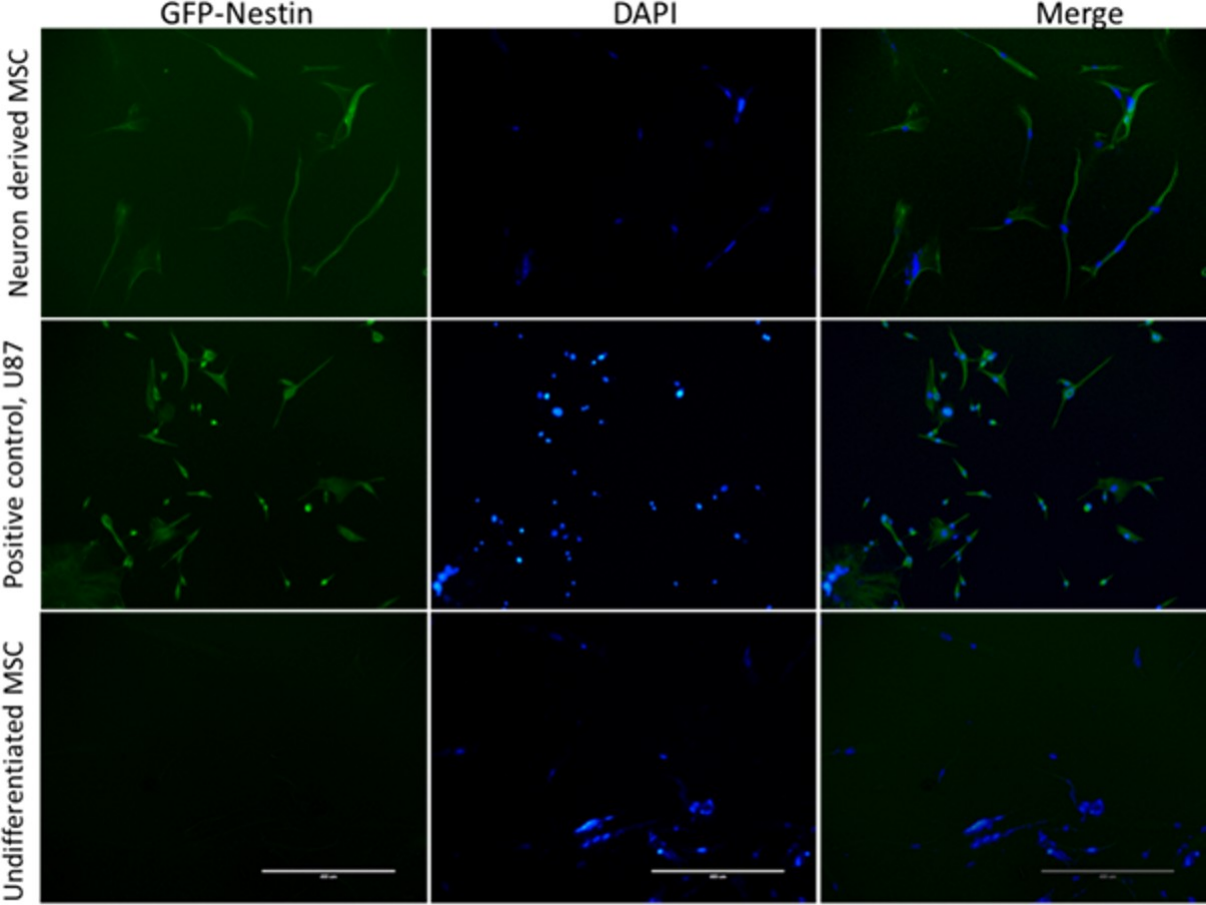
Nestin validation to confirm MSCs ability to differentiate into neurons. MSCs were differentiated into neurons through neuronal specific media. Top raw shows differentiated MSCs which are nestin, a neural positive marker (indicated by the green dye). Meddle raw shows positive control U87 (Neural cell line) which are also nestin positive, green dye. Bottom raw shows negative stain for nestin on undifferentiated MSCs (Indicated by the absence of color).

The isolated CVMSCs were also able to successfully differentiate into neurons "Fig 2 ". These findings confirm that the isolated cells are MSCs.

#### Characterization of HUVECs

All HUVECs derived from normal healthy women at passage 2 were more than 95% positive for HUVECs marker; CD31 as in "S1 Fig ".

### Cancer hallmarks

#### CVMSCs reduce malignant TNBC cells proliferation

**Fig 3:**
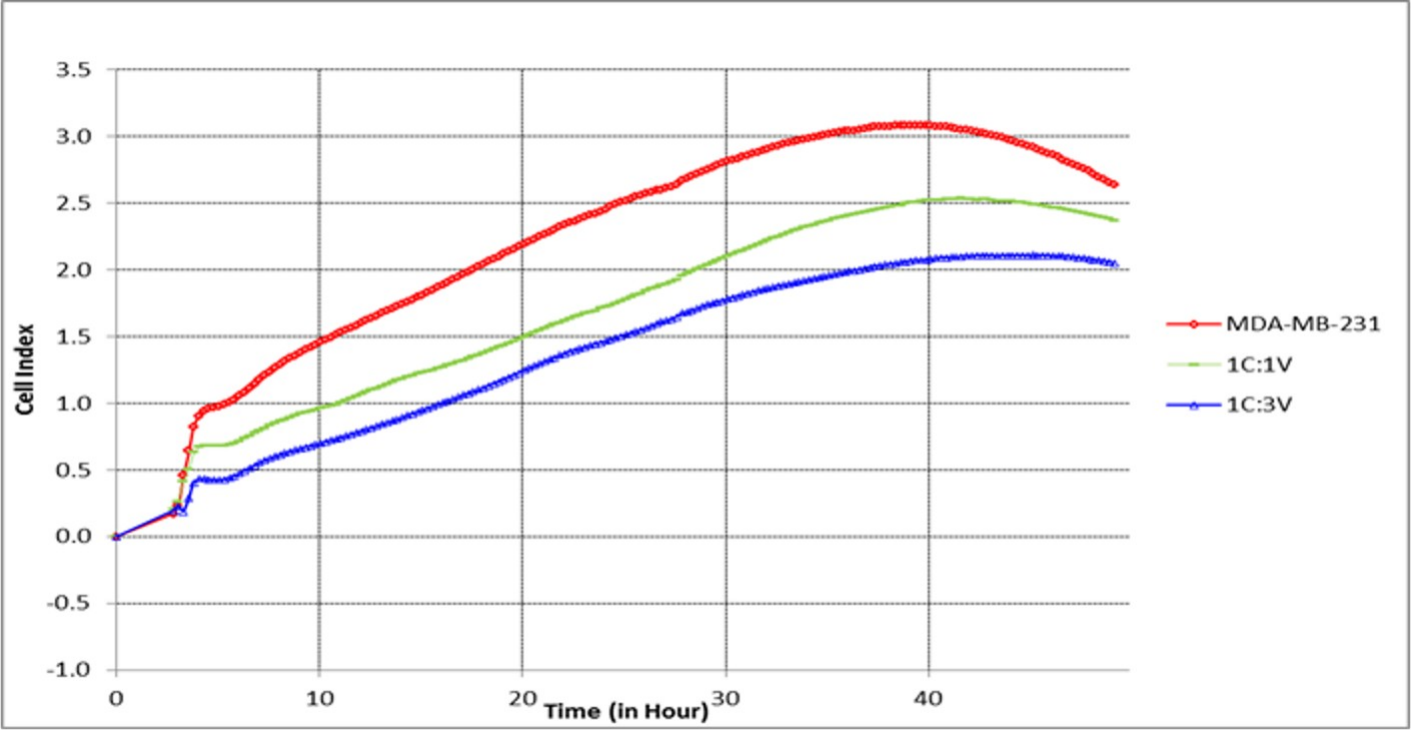
The effect of CVMSCs on MDA-MB231 proliferation. The effect of CVMSCs on MDA-MB231 cells proliferation was assessed *in vitro* over 50 hour. C is MDA-MB231, and V is CVMSC. MDA-MB231 cells were pretreated with CVMSCs through a transwell membrane. The reduction in the proliferative capacity of MDA-MB231 induced by CVMSCs was statistically significant (*P<0*.*0001)* compared to the control; untreated MDA-MB231). Each experiment was done in quadruplicate.

To investigate the effect of CVMSCs on the proliferative ability of MDA-MB231, MDA-MB231 cells were first pretreated with CVMSCs in a transwell membrane setting. Then, an xCELLigence proliferation assay was conducted on the treated MDA-MB231. The proliferation rate of the pretreated MDA-MB231 with high dose of CVMSCs (1 MDA-MB231:3 CVMSCs) was significantly lower than the control (un-treated MDA-MB231). The treated cancer cells with low dose of CVMSCs (1 MDA-MB231:1 CVMSCs) also had a reduction in the proliferation rate compared to the control (*P*<0.0001) as tabulated in Table 1 and depicted in "Fig 3 ". The MDA-MB231cells were also pretreated with conditioned media of CVMSCs with different ratios, prior to conducting the proliferation assay, but showed no effect (data not shown).

**Table 1:** The effect of CVMSCs on MDA-MB231 cells proliferation, in vitro, over 50 hour period (data represent a pool of 4 independent repeats).

Time (hh:mm:ss)	MDA-MB231		1C:1V		1C:3V		Statistical analysis
	Mean	SD	Mean	SD	Mean	SD	Time accounts for 63.19% of the total variance P<0.0001
00:00:00	0	0	0	0	0	0	
03:02:51	0.23	0.7	0.2	0.6	0.23	0.6	
05:48:10	1.02	0.6	0.7	0.6	0.4	0.5	
10:48:43	1.5	0.5	0.9	0.5	0.7	0.4	
23:50:09	2.4	0.2	1.7	0.25	1.4	0.2	
35:51:27	3.04	0.13	2.4	0.05	1.9	0.03	
47:52:47	2.74	0.04	2.4	0.02	2.0	0.01	

MDA-MB231 cells were pretreated with different rations of CVMSCs in a transwell membrane. The proliferative rate of the treated MDA-MB231 was monitored and compared to untreated MDA-MB231. C: MDA-MB231 and V: CVMSCs.

#### CVMSCs reduce malignant TNBC cells migration

**Fig 4:**
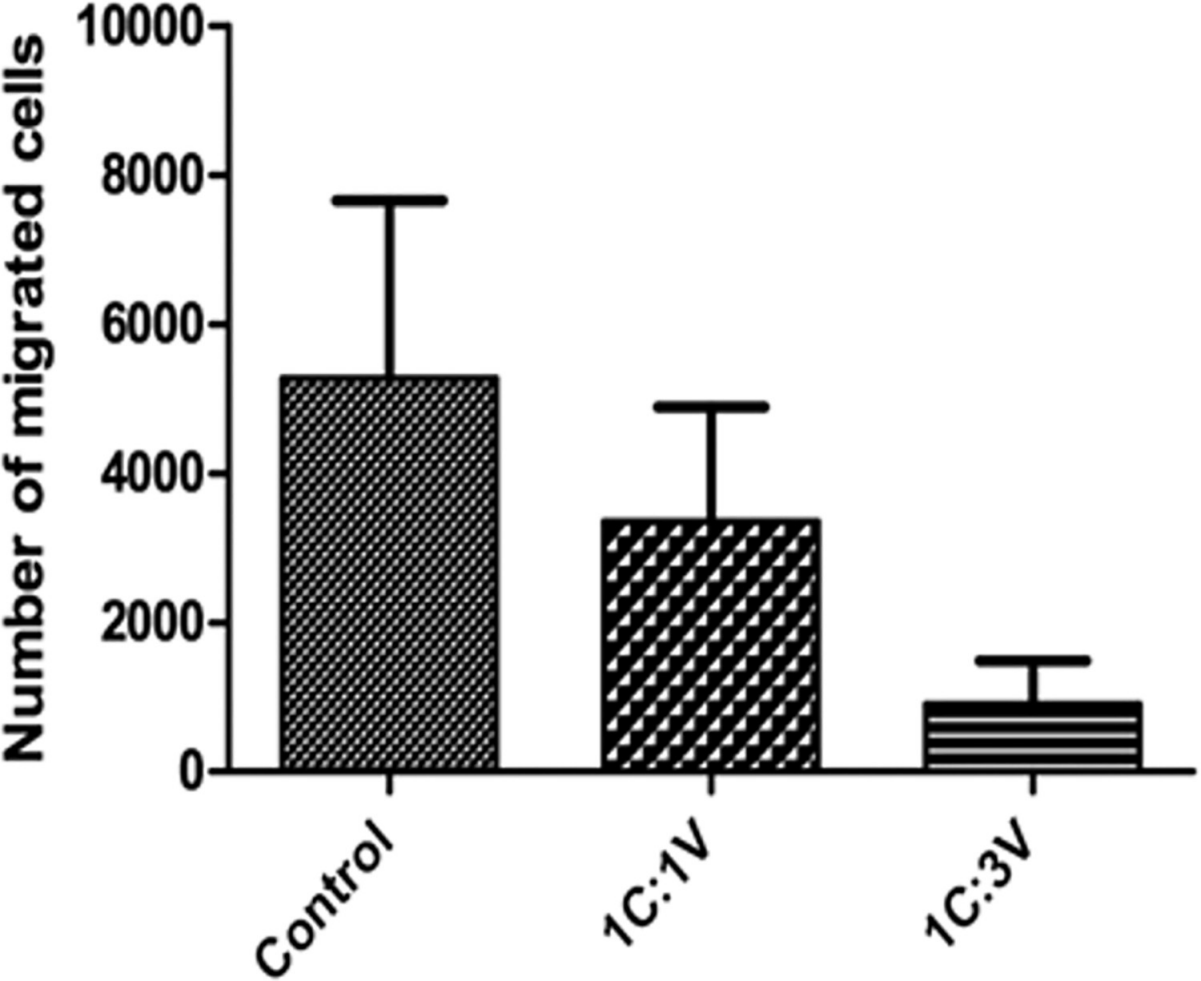
The effect of CVMSCs on MDA-MB231 cell migration using Boyden chamber migration assay. Where the control is C (MDA-MB231), and V is human placental chorionic villi derived MSC. The reduction in the migration response induced by CVMSCs was statistically significant (P = 0.037). Each experiment was repeated 3 times.

Before examining the migration ability of MDA-MB231, these cells were pretreated with CVMSCs through a transwell membrane. Then, a Boyden chamber migration assay was conducted on the treated cells. The migration capacity of the pretreated MDA-MB231 cells with CVMSCs either with high or low dose was significantly decreased compared to untreated cells *P* = 0.037 as can be seen in "Fig 4 ". MDA-MB231 cells were also pretreated with conditioned media of CVMSCs with different ratios, however this had no effect on their migration (Data not shown).

#### Tube network formation in vitro

**Fig 5:**
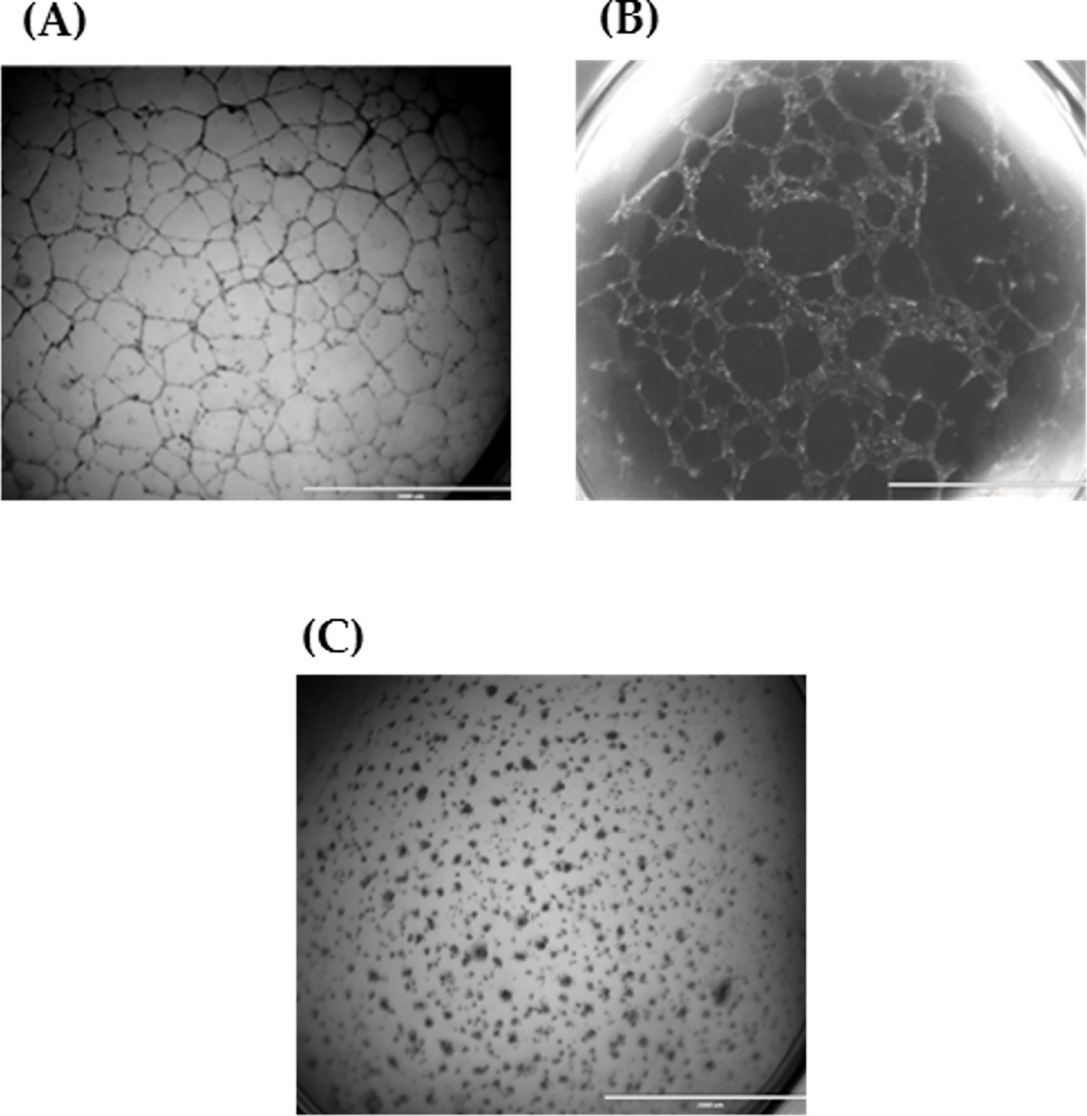
Tube formation of individual cell population. **(A)** HUVECs; **(B)** MDA-MB231 and; **(C)** CVMSCs on Matrigel with VEGF for 24h incubation. Tubes started to form following 14 hour of incubation. 2x magnification.

**Fig 6:**
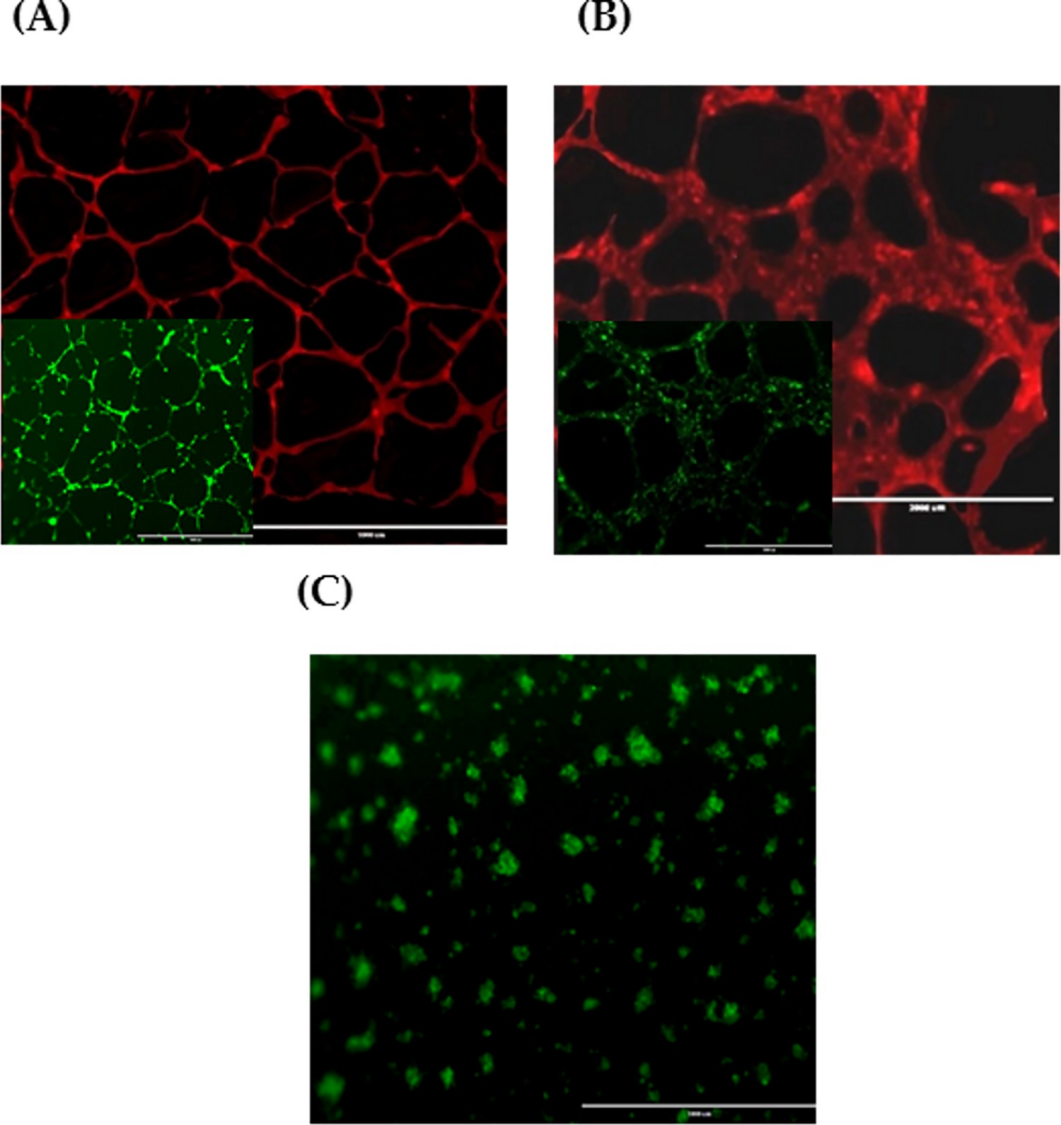
CD31 and Calcein AM staining of individual cell population. **(A)** HUVECs; **(B)** MDA-MB231 and; **(C)** CVMSCs. Images show both CD31 (red) and Calcein AM staining (green) for HUVECs and MDA-MB231. Whereas CVMSCs, show no expression of CD31. (2x magnification for CD31 and 10x magnification for Calcein AM). 2x magnification for **(C)**.

First, prior to creating the 3D tumour angiogenesis model of CVMSCs, MDA-MB231 and HUVECs co-culture, the tube formation capacity for each cell type was assessed individually. Tube formation of HUVECs, CVMSCs, and MDA-MB231 with the aid of VEGF on Matrigel is as shown in "Fig 5 ". HUVECs "Fig 5A " were used as a positive control to compare the tube development of MDA-MB231 and CVMSCs. While it could be observed that MDA-MB231 "Fig 5B " developed tubes similar to those of HUVECs, it is of the contrary that CVMSCs "Fig 5C " aggregated and failed to form any tube connections. The result of CD31 and Calcein AM staining of HUVECs, CVMSCs, and MDA-MB231 can be seen in "Fig 6 ".

#### CVMSCs inhibit tube formation of MDA-MD231

**Fig 7:**
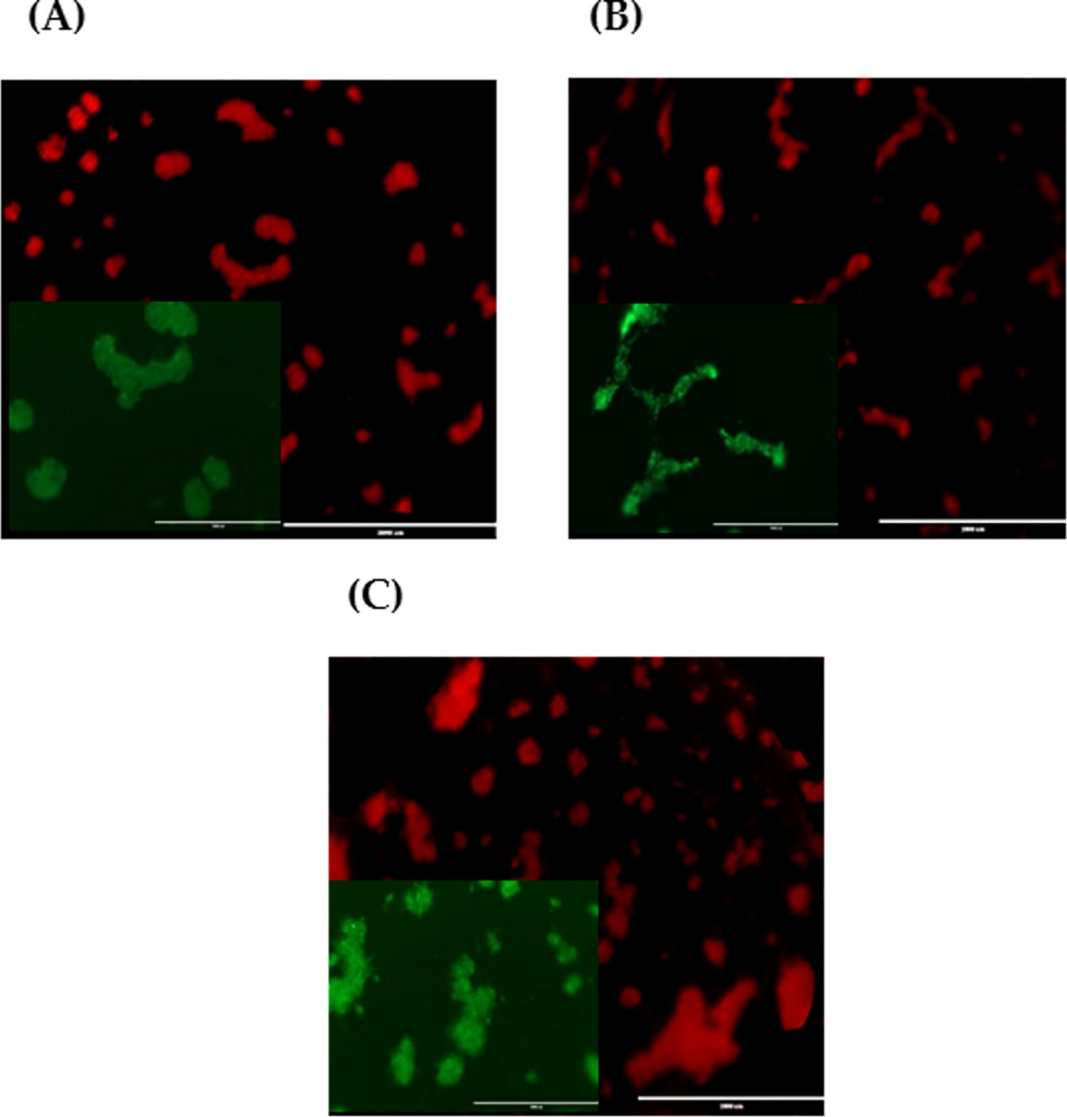
Tube formation of CVMSCs and MDA-MB231 co-culture at different ratios. **(A)** Co-culture 1 CVMSCs and 1 MDA-MB231; **(B)** Co-culture 1 CVMSCs and 2 MDA-MB231 and; **(C)** Co-culture 3 CVMSCs and 1 MDA-MB231 on Matrigel with VEGF for 24h incubation. Images show CD31 (red) and Calcein AM staining (green) staining. 10x magnification for Calcein AM and 2x magnification for CD31.

Second, an in vitro 3D tube formation assay was conducted using a co-cultured CVMSCs with MDA-MB231. This is done to assess the effect of CVMSCs on MDA-MB231 ability to form capillary-like structure. The ability of MDA-MB231 to form capillary-like structure (as seen in "Fig 6B ") was significantly lowered when they were co-cultured with CVMSCs "Fig 7 ". Tube formation was inhibited regardless of the cell ratio [1:1, 1:3, and 2:1 (MDA-MB231:CVMSCs)]. Although the experiment was carried out at ratios 1:2 and 3:1, the results were recorded despite not being depicted.

In a separate experiment, MDA-MB231cells were also pretreated with CVMSCs conditioned media at different concentrations, but this had no effect. Therefore, in the present study the followed protocol for angiogenesis assay includes mixed-population monolayers and feeder layers in flasks [22, 23].

#### CVMSCs reduce/inhibit tube formation of MDA-MB231 and HUVECs

**Fig 8:**
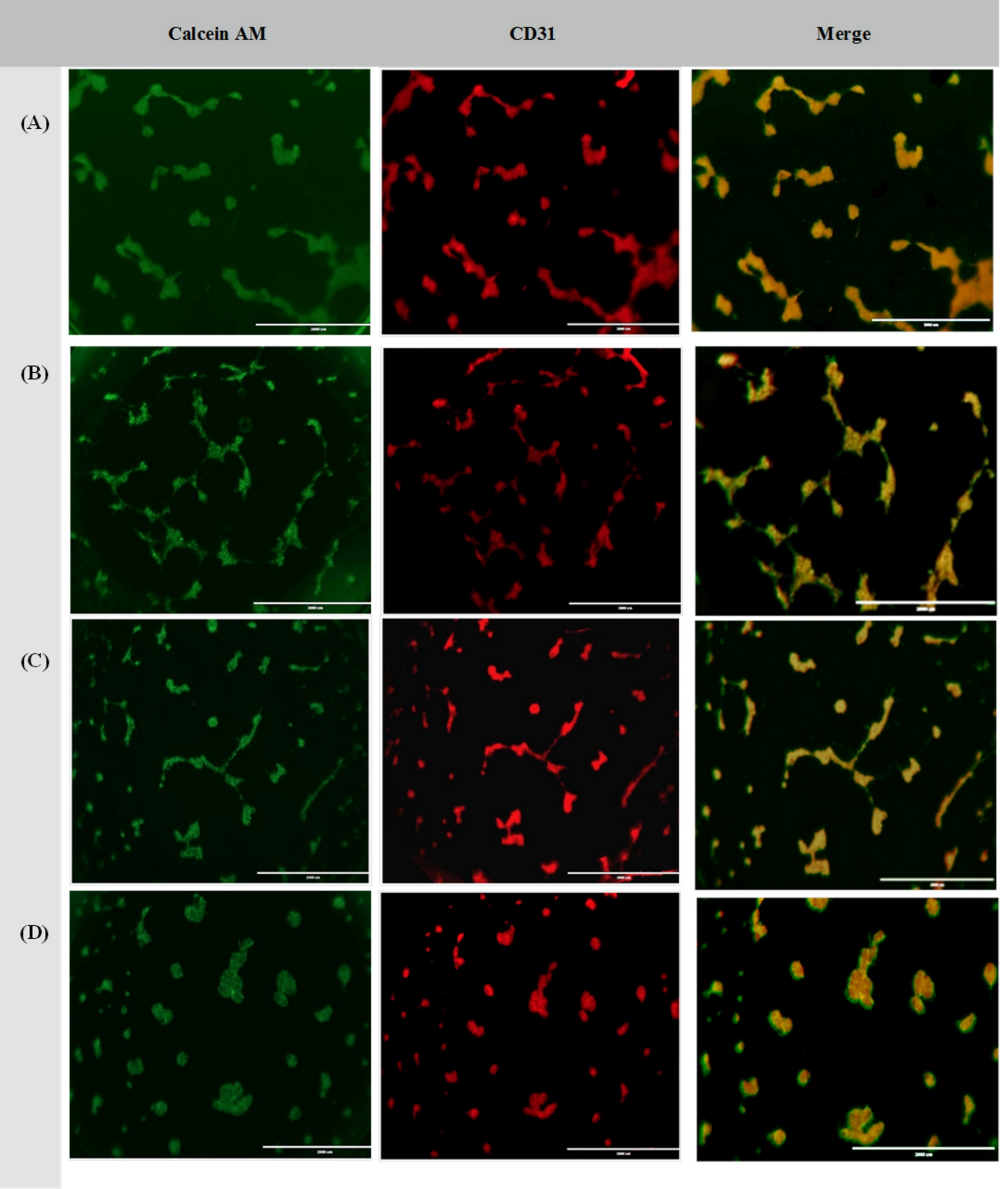
Tube formation of CVMSCs, MDA-MB231 and HUVECs co-culture at different ratios. **(A)** Co-culture of 1 HUVECs: 1CVMSCs and 1 MDA-MB231; **(B)** Co-culture of 1 HUVECs: 1 CVMSCs and 2MDA-MB231 and; **(C)** Co-culture of 1 HUVECs: 2 CVMSCs and 1 MDA-MB231 and; **(D)** Co-culture of 1 HUVECs: 3 CVMSCs and 1 MDA-MB231 on Matrigel with VEGF for 24h incubation. Images show CD31 (red) and Calcein AM (green) staining or both overlay at 10x magnification.

Third, the in vitro 3D tube formation assay was repeated using a co-cultured of CVMSCs, MDA-MB231 along with HUVECs. This is done to assess the effect of CVMSCs on MDA-MB231 ability to form capillary-like structure in the presence of HUVECs. The results are as depicted in "Fig 8 ". Even with the presence of HUVECs, the formation of a capillary-like structure by MDA-MB231 was still being inhibited by CVMSCs, regardless of the ratio. This evidence is further supported by the result of complete inhibition of tube formation when the ratio of CVMSCs is increased to 1:1:3 (HUVECs: MDA-MB231: CVMSCs) as depicted in "Fig 8D ". The inhibition of the tube formation is referred to CVMSCs because it was not detected, unless CVMSCs are added.

#### Angiogenic index analysis of the treated MDA-MB231 with CVMSCs

**Fig 9:**
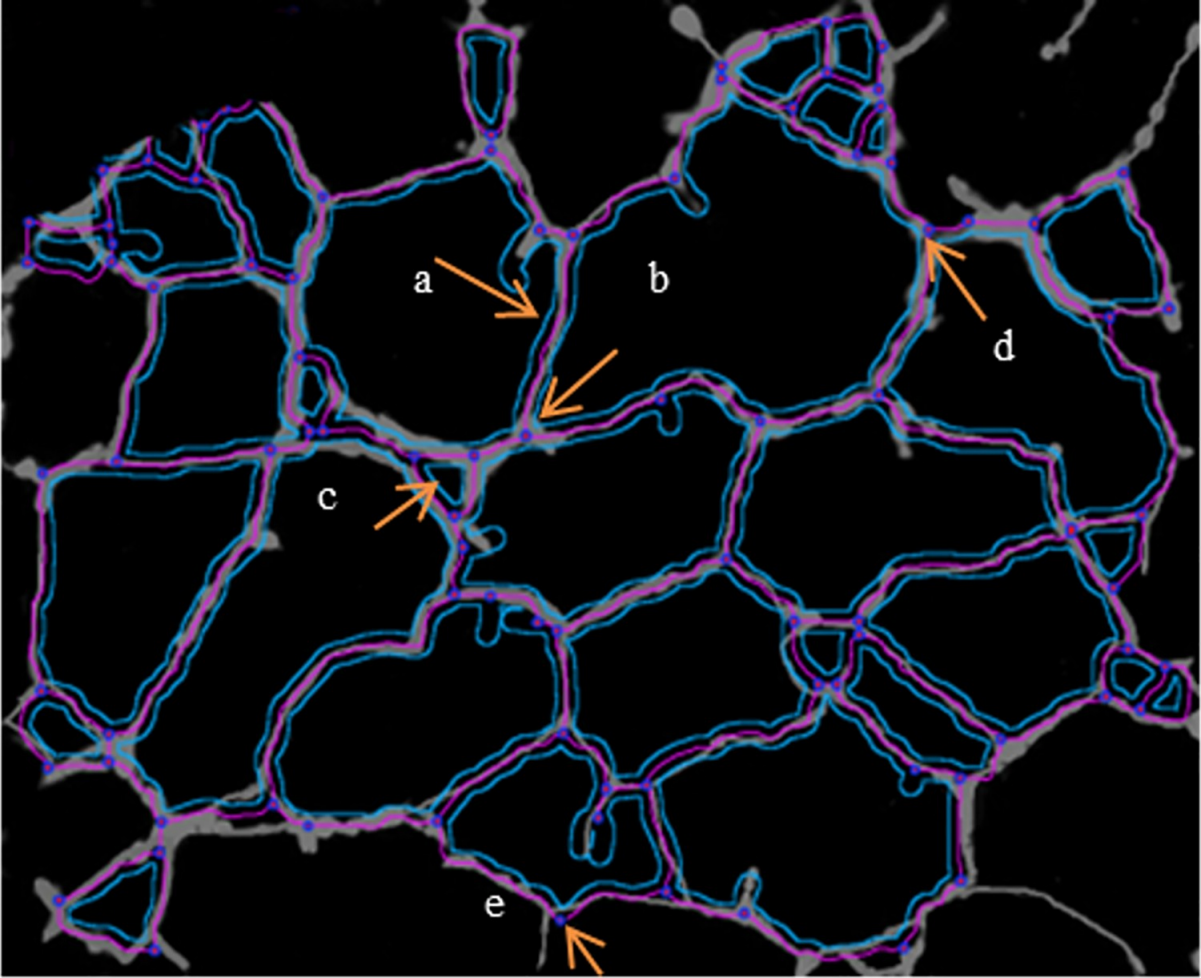
The detection of constitutive elements of the network—Summary. A tree composed from master segment (a); associated by master junction (b); delimiting the meshes (c); junction (note that this pointed junction is composed by several nodes) (d); node identified as three neighbors (e).

**Fig 10:**
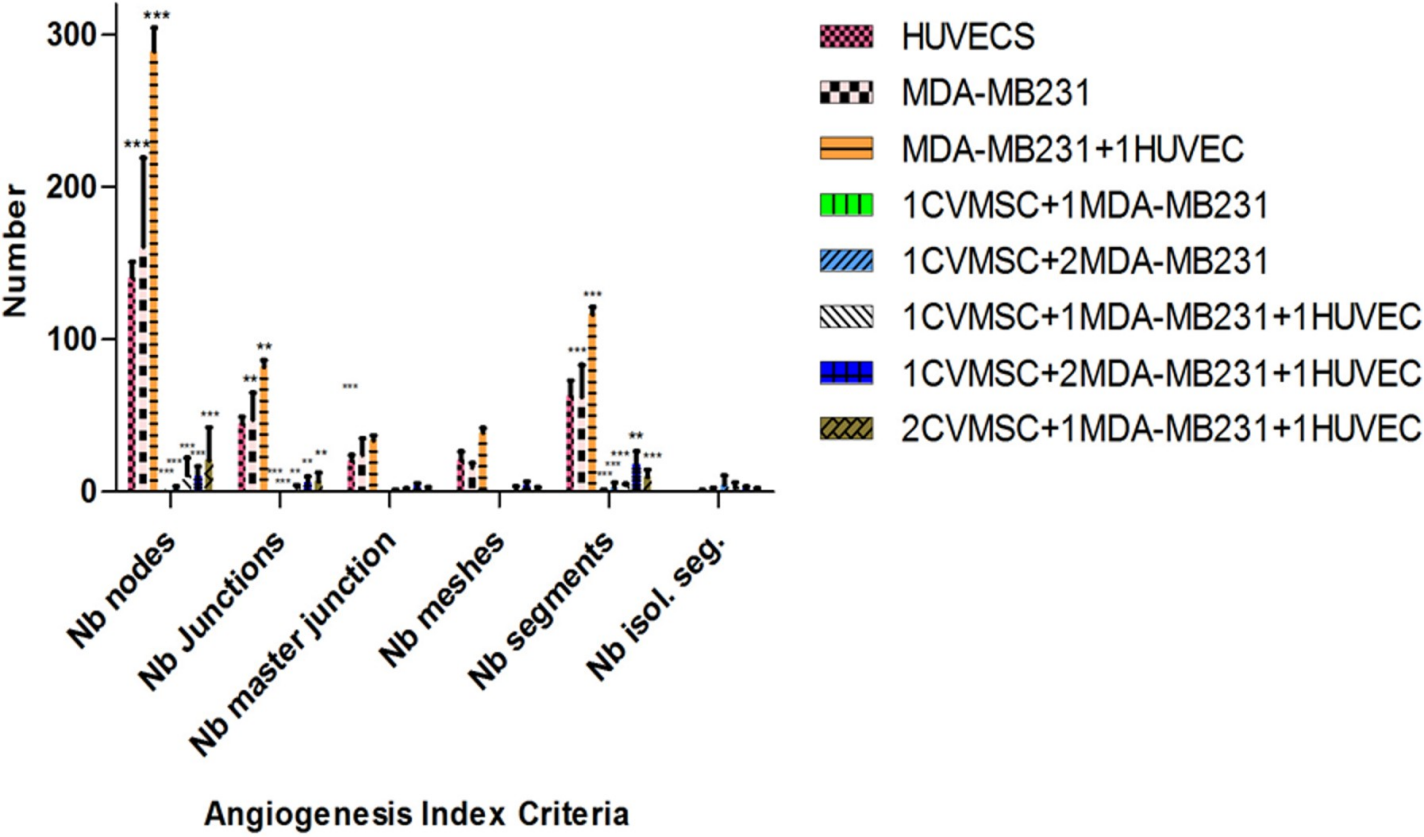
Statistical computation of angiogenic parameters using angiogenesis analyzer macro connected image J software. The bars show a comparison between untreated MDA-MB231 and treated MDA-MB231 with different ration of CVMSCs in the presence of HUVECs or not. Nb = number. All the groups were analyzed by Two-way ANOVA and each group was compared to the positive group (HUVECs). HUVEC = human vascular endothelial cells, CVMSC = of human placental chorionic villi derived MSCs.

Finally, to examine the angiogenic morphology systematically, we measured six angiogenic indexes of MDA-MB231 cells treated with CVMSCs at different ratios with or without HUVECs. The measurement were conducted using the Angiogenic Analyzer macro (Image J software 1.50 version) and they included; nodes, junctions, master junction, meshes, number of segments, and number of isolated segments "Fig 9 ". The treated MDA-MB231 with CVMSCs with/out HUVECs showed a loss of network or the cells formed clusters rather than tubes, therefore, it is difficult to identify the main angiogenesis index criteria such as the number of extremities, number of master segments, number of branches and the length of the tubes. The Angiogenic Analyzer identifies vessel networks and evaluates the cells vascular organization. To assess the anti-angiogenic effects of CVMSCs, the Analyzer acquires a quantitative evaluation of the vessel network by extracting distinguishing information of the angiogenic images. "Fig 10” shows the angiogenesis index criteria on treated and untreated MDA-MB231. Both HUVECs and untreated MDA-MB231 showed no isolated segments, whereas MDA-MB231 co-cultured with CVMSCs showed the highest number of isolated segments. The co-cultured of MDA-MB231 cells with CVMSCs with/out HUVECs reduced or inhibited angiogenic parameters including number of nodes, junctions, and meshes as compared to control cells (MDA-MB231 or HUVECs) P<0.01. In this section, only 1:1, 1:2 and 2:1 ratios (MDA-MB231:CVMSCs) with/out HUVECs were analyzed, because 1:3 showed no tube at all.

### The reduction of cytokines and chemokines expression in pre-treated CVMSCs with MDA-MB231

**Fig 11:**
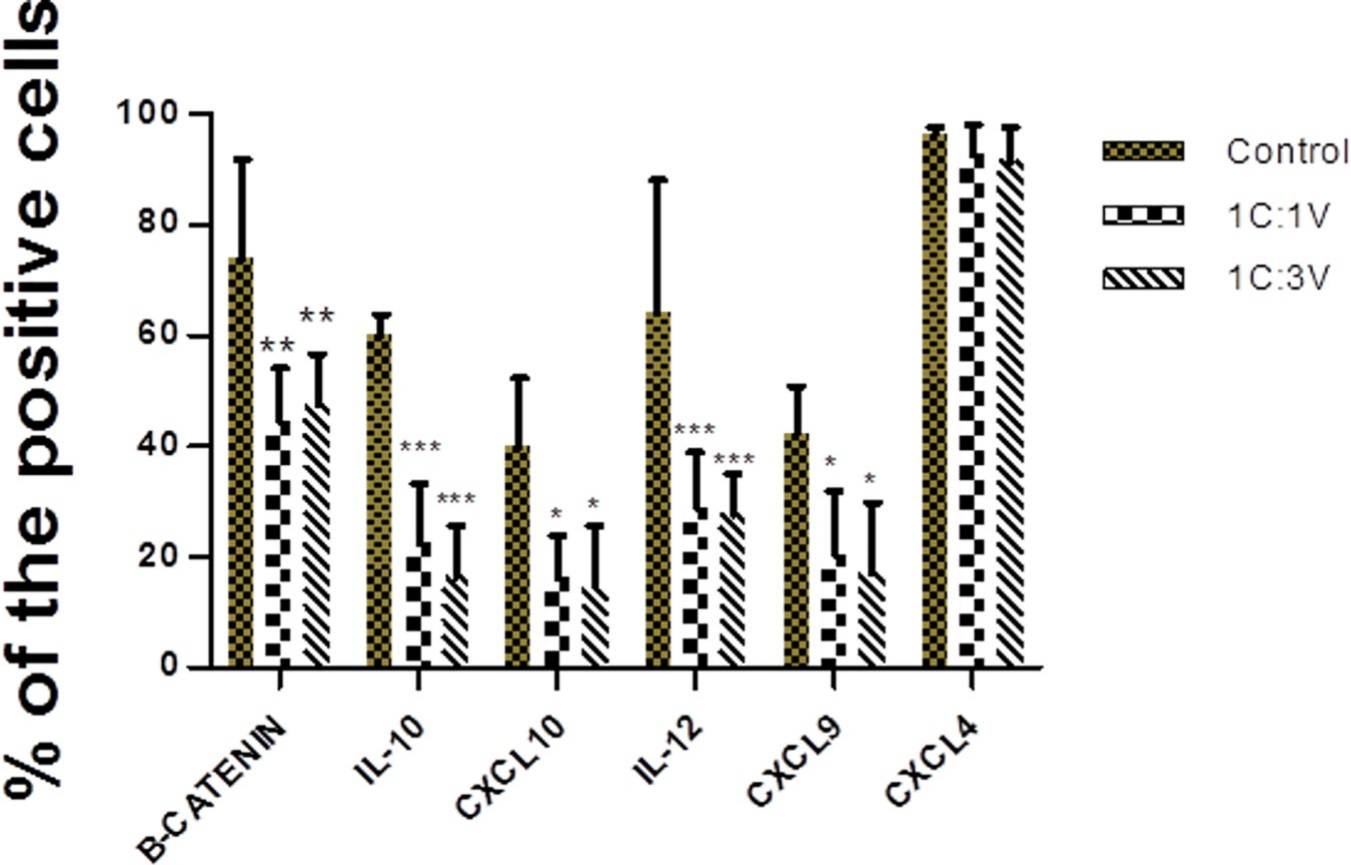
Expression of cytokines and chemokines in pre-treated CVMSCs with MDA-MB231 through a transwell membrane. Two-way ANOVA analyzed the two treated groups; 1:1 and 1:3 and each group was compared to the control. The control is CVMSCs without any treatment. C is MDA-MB231, V is CVMSCs. CVMSCs = human placental chorionic villi derived MSCs.

CVMSCs were treated with the MDA-MB231 cells, then the expression level of a number of breast cancer associated cytokines and chemokines was measured via flow cytometry. This was done to examine if malignant cells will have a ‘malignant effect’ on these cells. The expression level of IL-10, IL-12, CXCL9 and CXCL10, in CVMSCs treated with MDA-MB231 was significantly lower compared to the control (untreated CVMSCs) (as seen in "Fig 11 ").

## Discussion

The revelation that MSCs are enlisted into the tumor’s microenvironment has prompted an extensive investigation on the role of these cells in the process of tumourigenesis, and specifically tumor-associated angiogenesis. Interestingly, studies on this subject matter are reporting contradicting results, with some investigators showing that MSCs enhance the development of the tumors [24–26]. While others reporting that, MSCs inhibit tumor growth and development [23, 27, 28]. With each side implicating different mechanisms of action that include; chemokine signaling, immune modulation, as well as vascular support. Subsequently, researchers have attempted to explain these contradictory reports on MSCs and their immunomodulatory effect in the maintenance of tumours. For example Waterman et. al. (2010) reported that MSCs can be polarized into two phenotypes; a pro-inflammatory “MSC1” and an immunosuppressive “MSC2” phenotype [29]. When MSCs were primed with TRL4, they increased the secretion of pro-inflammatory factors [29, 30]. Whereas, TLR3 priming, caused MSCs to increase the secretion of factors with immunosuppressive properties [29, 30]. Thus, weather the administered MSCs are anti or pro-tumour depends on the phenotype. Whereas, Klopp et al, (2011) compared and analyzed the methods used in published studies, and postulated that the timing of MSCs administration into the tumour microenvironment is critical factor in determining whether these cells will adapt an anti or pro-tumor effect [31]. Thus, in this study, we aimed to investigate the effect of the placental derived MSCs, CVMSCs, on breast cancer hallmarks, and whether these cells are anti or pro-tumor, and if they could be utilized in a ‘cell-based therapy approach’ for breast cancer.

The placenta, with its diverse cell population, plays a central role in maintaining a pregnancy. One of its essential functions, besides providing oxygen and nutrition, is to provide a gestational/maternal immune tolerance via immune modulations. This prevents the mother from generating an immune response against the fetus, which is crucial for successful completion of the pregnancy [32]. Thus, it is believed that placenta derived cells, including MSCs, have an intrinsic immunomodulation capacity. In general, the immunomodulatory role of MSC is well-documented [33–36], and it’s thought to be mainly through the regulation of T-reg cells, which can suppress both the migration and proliferation of peripheral blood mononuclear cells and inhibit natural killer cells and cytotoxic T- cell function [36]. For placental derived MSCs, in particular, they are known to possess anti-inflammatory, pro-angiogenic, cytoprotective and regenerative properties initiated by paracrine factors within its microenvironment [37, 38]. However, the defined properties of placental MSCs sub-populations from the fetal vs. maternal side of the placenta are still vague. Studies are reporting some dissimilarities between placental MSCs derived from different parts of the placenta. For example it was reported MSCs derived from the fetal side of the placenta have stronger immunomodulatory effect than those driven from the maternal side [39]. Therefore, in this study we used one uniform placental MSCs population of fetal origin, CVMSCs. This subpopulation of placental MSCs were isolated based on a published protocol and were verified to meet the accepted MSCs criteria.

Following successful CVMSCs isolation and characterization, the investigation commenced by examining the effect of CVMSCs on MDA-MB231, a TNBC cell line, proliferation, and migration. MDA-MB231 cells were treated with CVMSCs via direct contact using a transwell membrane. Then, a real time cell proliferation assay utilizing the xCELLigence system and Boyden chamber migration assay were conducted. Data from both assays revealed that CVMSCs significantly reduced MDA-MB231 cells proliferation as well as migration. Next, a 3D tube formation assay was conducted to examine the vasculogenic capacity of MDA-MB231 cells with and without CVMSCs co-culture. First, the vasculogenic capacity of MDA-MB231 cells was evaluated independently of CVMSCs or endothelial cell-associated angiogenesis. In this study, it was shown that MDA-MB231 cells, alone, were capable of developing vascular networks on Matrigel, in line with vasculogenic behaviour of this type of tumour cells [40]. Then, the vasculogenic capacity of MDA-MB231 co-cultured with CVMSCs and HUVECs was evaluated again, using the same assay. Data showed that CVMSCs inhibited MDA-MB231 cells tube formation even with the addition of HUVECs. Moreover, the inhibition of MDA-MB231/ HUVECs angiogenesis by CVMSCs was dose-dependent as by increasing the concentration of CVMSCs, a complete inhibition of angiogenesis was obtained.

The results from the angiogenic assay are consistent with the results from the proliferation and migration data, as well as the cytokine analysis of CVMSCs assay. When CVMSCs were pretreated with MDA-MB231, there was a markedly reduced expression of IL-10, IL-12, CXCL9 and CXCL10 on CVMSCs. This indicates that CVMSCs, not only have the ability to affect both MDA-MB231 cells and HUVECs behavior, but also CVMSCs have the ability to response to signals from the MDA-MB231 cells, and adapt its cytokine profile accordingly, in order to modulate or halt the proliferation, migration and tube formation of MDA-MB231, i.e. inhibit tumor progression. One of the down regulated cytokines was IL-10, CVMSCs that were treated with MDA-MB231 had a significantly lower IL-10 levels compared to untreated CVMSCs. This could have played a role in the significant tube formation inhabitation, when CVMSCs were co-cultured with MDA-MB231/HUVECs. A number of studies have showed that IL-10 reduces the synthesis level of VEGF, TNF-α, or MMP-9, which leads to a prevention of angiogenesis associated with the growth of the tumour; such as melanoma and prostate cancer [41, 42]. On the other hand, Silvestre et al. (2000) have stated that angiogenesis in the ischemic hindlimb was significantly increased in IL-10(-/-) compared with IL-10(+/+) mice [43]. Another down regulated cytokines were CXCL9 and CXCL10, members of the CXC cytokine family. This family of cytokines are known for their ability to behave in a different manner in regulating angiogenesis [44]. It is known that several members of the CXC chemokine are effective promoters of angiogenesis, whereas others inhibit the angiogenic process [45]. The data from this study could suggest that CXCL9 and CXCL10 could play a role in tumor angiogenesis, as down regulation of which on the CVMSCs, have affected MDA-MB231/HUVECs tube formation.

Angiogenesis or formation of new blood vessels from pre-existing ones, is a process that supports the development of tumors and increases the incidence of metastasis. Thus, therapeutic approaches that employ anti-angiogenic agents are now being considered for TNBC [46]. The reports on the role of MSC in angiogenesis are are inconsistent. MSCs are often believed to be an angiogenic agent [47–50], and it is believed that they do that through the release of trophic factors [51]. For placental MSCs, it was reported that MSCs from the fetal side, but not maternal side, had the ability to stimulate *in vitro* angiogenesis [52]. On the other hand, other studies are reporting an anti-angiogenic effect of MSCs, [53–55]. Again for placental MSCs, in this study, it was demonstrated that that MSCs from the fetal side, CVMSCs, inhibited the independent and endothelial cell-associated vasculogenic capacity of MDA-MB231 cells. The inconsistency in the results from this study and other studies could be attributed to a number of factors. These factors include, the type of MSCs sub-population used, the type of cancer cells being investigated, systems of in vitro culture used, technicalities related to the *in vitro* assay conduction i.e. ratio of cells, angiogenesis-stimulator factor used, route of administration …etc. Never the less, as far as we know, this is the first study showing the effect of CVMSCs on MDA-MB231 induced angiogenesis. Clumping of cells or diminishing length of the tube may be translated as a defence mechanism against tumour progression or angiogenesis. The findings from this study could provide a ground work for a CVMSC-based, anti-angiogenic therapy for TNBC and perhaps other forms of cancers.

## Materials and methods

### Tissue collection

For CVMSCs and HUVECs isolation, placentae were collected with the approval from the Institutional Research Board Committee-King Abdullah International Medical Research centre, Riyadh, Saudi Arabia. All placentae were collected following informed, written consent from conscious fully aware mothers, at the King Fahad Hospital, King Adulaziz Medical City, Riyadh, Saudi Arabia. The collected placentae were from healthy, clinically uncomplicated pregnancies with normal macroscopic morphology.

### Isolation and culture of CVMSCs

Human placentae were obtained from uncomplicated pregnancies following normal vaginal delivery at around 38 to 40 weeks of gestation. The placentae were used within 2 h of delivery. Isolation of CVMSCs was carried out as previously described [56]. The isolation was performed on the fetal side, which has the umbilical cord insertion. The chorionic villi tissue was dissected and then washed thoroughly using sterile Hank's Balanced Salt Solution (HBSS).

### CVMSCs were validated using:

#### Flowcytometric analysis of MSC surface markers:

Flow cytometry analyses were carried out to confirm positive markers for MSCs including CD90 (IM1839U), CD105 (A07414), CD144 (1FAB9381p) and CD166 (A22361) (All these markers were purchased from Beckman Coulter, USA). In addition, two negative markers HLA-DR (335830) and CD14 (345784) were tested (purchased from BD, USA). Experimental testing started with cells’ preparation through harvesting of live cells with TrypLE Express Enzyme (Gibco, #12604013). Then, cells were washed with Dulbecco's phosphate-buffered saline ((PBS), (Gibco, #14190250)). Later, cells re-suspended in FACS buffer (0.5% Bovine serum albumin (BSA), 0.5% Fetal bovine serum (FBS) in 1xPBS). Fixation was done at two steps: 50% methanol and 100% methanol for 20mins. Cells then were re-suspended in cold blocking buffer (0.5% BSA and 2% FBS in 1x PBS) for 20 min. After that, we incubated the designated antibodies with cells suspended in cold FACS buffer for 35 min. Washing step was performed with cold FACS buffer just prior to flow cytometry experiment. Flow cytometry detection was performed through gating 10,000 cells using FACS Canto II machine, BD.

#### Differentiation potential

Induction of MSCs differentiation was processed with mechanical detachment of MSCs enzymatically using TrypLE Express Enzyme (Gibco, #12604013). Then, cells were suspended in special Dulbecco's Modified Eagle Medium (DMEM) neuronal specialized media. This special media contained 50% (Gibco, USA, #22320–022), 50% Neurobasal (Gemini, NeuroPlex #600301), B-27 Supplement minus Vitamin A (Gibco, #12587010), Antibiotic-Antimycotic (Gibco, USA, #15240062), 5% FBS (Gibco, USA, #10439024) and GlutaMAX Supplement (Gibco, USA, #35050061). The MSCs were maintained in the neuronal media for two weeks. After the detection of neuronal cells morphology under the microscope, the Nestin stain can be assayed. Nestin is considered a neural marker. U87 MG which is a brain cancer cell line (ATCC- HTB-14, USA) was used as positive control for Nestin stain. Briefly, Immunocytochemistry Protocol was performed with methanol fixation for 20 min. Then, cells were permeabilized by 0.1% Triton x-100 in PBS. Cellular blocking was done using 0.5% BSA and 2% FBS in 1x PBS. Next, primary antibody dilution was prepared in 1% normal serum and 0.01% tween. Antibody mixture was incubated for 2 hours. At that point, secondary antibody with FITC was prepared and incubated with cells for 30 min. Plate was imaged at EVO- auto FL microscope.

### Isolation and culture of human umbilical vein endothelial cells (HUVECs)

The cord was rinsed in PBS several times until it is clear of blood and the vein was identified in order to insert the feed needle. Using a sterile syringe, several changes of PBS were flushed through the vein to remove any clots and blockages. 20 ml of M199 (Invitrogen, Saudi Arabia) (without FBS or Penicillin or Glutamine) media was injected into the vein before 20ml filtered collagenase II (210u/ml in PBS) was added to the cord for 15 min at 37°C in a humidified atmosphere containing 5% CO_2_. A pale-yellow substance was then collected in the collection syringe. The cord was then flushed with media (M199+20% FBS+2% Penicillin/Streptomycin+1% Glutamine) for several rinses until the fluid in the collection syringe appears clear. The collected suspension was centrifuged at 1000 rpm for 10 minutes. A maximum of 1 x 10^5^ cells was seeded in a T25 flask. At 80% confluence or after 7 days whichever is earlier, the cells were characterized using flow cytometry (as mentioned above) with HUVECs positive markers; CD31 (R&D system, Saudi Arabia).

### Cell lines culture

The MDA-MB231 TNBC cell line was used for this study (ATCC, USA). MDA-MB231 cell line is characterized by triple-negative phenotype and are enrichment for markers associated with the epithelial-mesenchymal transition and the expression of features associated with mammary cancer stem cells (CSCs), such as the CD44+CD24-/low phenotype [57]. MDA-MB231 was cultured in DMEM-F12 medium containing 10% qualifies NZ FBS (Invitrogen, Saudi Arabia), 100 μg/ml of L-glutamate, 100 μg/ml streptomycin and 100 U/l penicillin (Invitrogen, Saudi Arabia). When the cells reached 80% confluence, the MDA-MB231 cells were harvested by trypsinization and centrifugation at 500 xg for 10 minutes and 200 xg for 5 minutes respectively.

### Cancer function assays

Three hallmarks of cancer have been studied, including proliferation, migration and angiogenesis. Before performing these assays, MDA-MB231 cells were treated in three methods: 1) Conditioned Media (CM) of CVMSCs; 2) **D**irect contact with the CVMSCs through a transwell membrane; 3) Direct co-culture involves two or more cell types of interest being overlaid upon one another (HUVECs, CVMSCs and MDA-MB231). The latter was only used for angiogenesis, because the former systems showed no effect. In this system cellular interactions occur through direct cell–cell contact and soluble factors [22]. The main aim of this system is to detect any change on the formed tubes.

#### Preparation of conditioned media of CVMSCs

Cells were grown in their specific medium DMEM-F12 containing 10% MSC qualified Australian FBS (Invitrogen, Saudi Arabia), 100 μg/ml of L-glutamate, 100 μg/ml streptomycin and 100 U/l penicillin (Invitrogen, Saudi Arabia) in 75 cm^2^ flasks. When the cells reached 75–80% confluence, the **conditioned media (**CM) was harvested. Therefore, CM was centrifuged at 2000 x g at 4°C for 30 min, and stored at -21°C until use for proliferation, migration and angiogenesis.

#### Transwell membrane assay for the treatment of TNBC cell with CVMSCs

**Fig 12:**
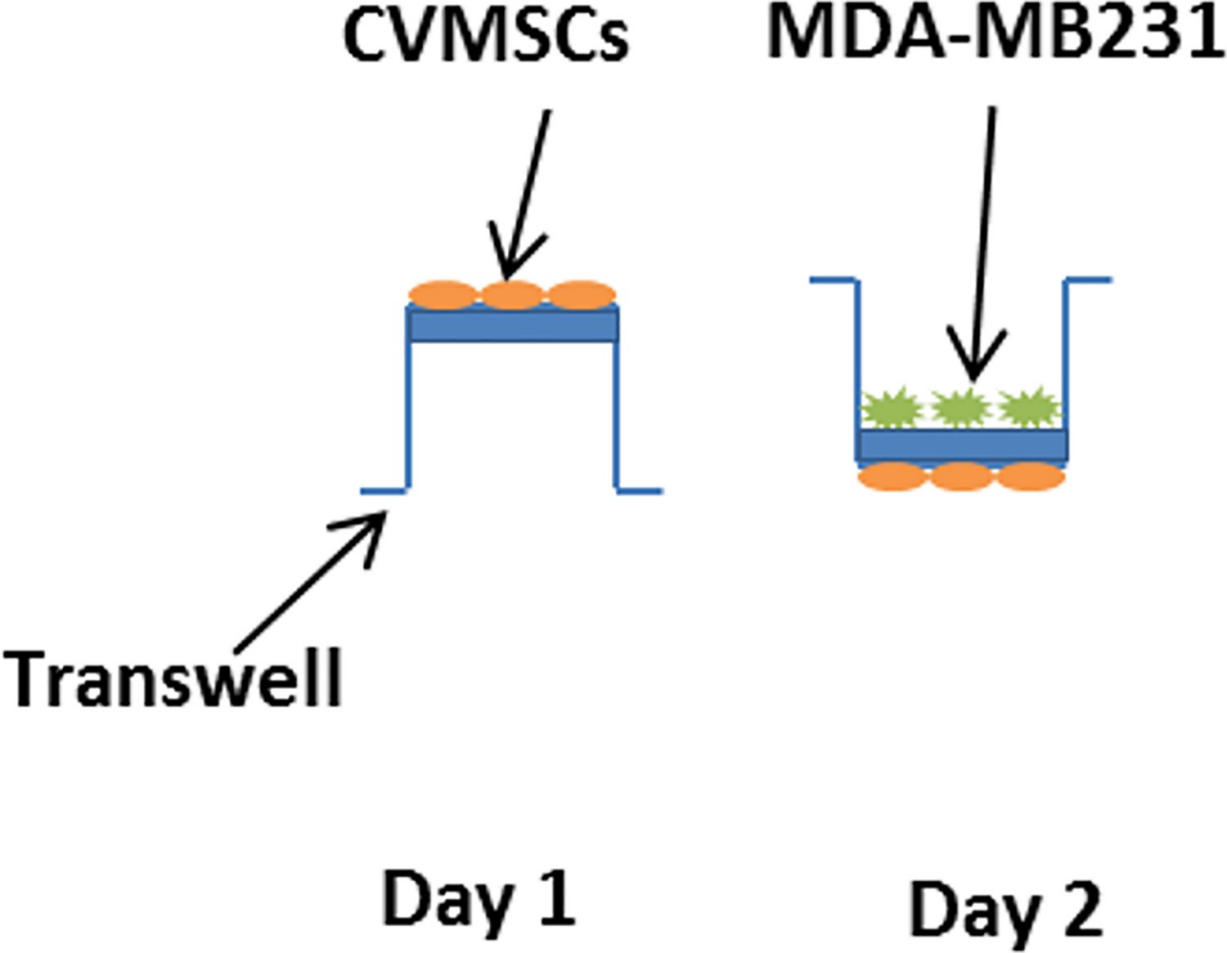
A direct contact between CVMSCs and MDA-MB231 through a transwell membrane. CVMSCs were incubated overnight on the underside of the transwell. After 24 h the transwell was flipped over and MDA-MB231 cells were added on the upper chamber of the transwell and incubated for 48hr.

This experiment was designed to test whether CVMSCs seeded in the bottom chamber (a transwell membrane) express factors that attract MDA-MB231 cells on the top chamber of the same insert "Fig 12 ". On the first day, 1 x 10^5^ cells of CVMSCs were incubated overnight on the bottom chamber of the transwell. After 24 hours, the transwell then was flipped over and inserted in a 6-well plate. Therefore, MDA-MB231 cells were added on the top chamber of the transwell membrane at the same density or as required (0.4 um membrane insert, Greiner Bio-one, USA) and incubated for 48 hours. Finally, only MDA-MB231cells were collected after trypsinization and used for the next experiments (proliferation and migration). The ratios of MDA-MB231 to CVMSCs were 1:1 and 1:3.

#### Proliferation assay

The rate of MDA-MB231 cell proliferation was monitored in real time using the xCELLigence system (xCELLigence RTCA DP—ACEA Biosciences Inc.). The RTCA instrument is composed of a RTCA impedance analyzer, a 16-well disposable electronic microtiter plate (E-Plate), the RTCA station, which accommodates the E-Plate and maintained under tissue culture incubator, and a computer with RTCA software for controlling the system operations. 50 μL of cell culture medium was added to each well for the impedance background measurement. After adding the control cells (only MDA-MB231) and test cells which are MDA-MB231 cells treated with CVMSCs, the final volume was 200 μL. The E-Plates were incubated at 37°C with 5% CO_2_ and monitored on the RTCA system at 5-minutes intervals for up to 50 hour. The ratio of MDA-MB231 to CVMSCs was 1:1 and 1:3.

To facilitate the statistical assessment of the outcomes, each experiment was carried out at least in triplicate with condition as recommended in the technical manual of xCELLigence.

#### Boyden chamber migration assay

6-well polycarbonate inserts (8 μm; Greiner Bio-One, USA) were loaded in 6-well plates; filled with the culture medium of TNBCc (DMEM with 30% FBS) for Transwell migration assay. MDA-MB231 treated with CVMSCs and untreated MDA-MB231 at 1×10^5^ cells/well were cultured in serum-free medium and loaded into the plate well insert (2ml/well) until they adhered to the well. Subsequent to being cultured at 37°C for 24 hour, the cells remaining on the upper surface of the membrane were wiped away with a cotton tip applicator. The migrated cells on the lower surface of the membrane were fixed with 4% paraformaldehyde and stained with 1% crystal violet (sigma, USA). Images in different random fields were captured for quantification using light microscopy.

#### Matrigel preparation and tube formation assay

Since Matrigel solidifies at temperature above 25°C, the working temperature used to coat the wells was at cool room temperature (RT). 50 μl of Matrigel was used to coat the 96-well plates evenly and was incubated overnight at 37°C in 5% CO_2_ to solidify. Working density was set at 5 x 10^3^ and 20 x 10^3^ cells/well with 50 ng/ml of vascular endothelial growth factor (VEGF) (Corning, USA) as angiogenesis-stimulator factor for angiogenesis. HUVECs only and CVMSCs only were cultured in their basic culture media as control groups. Tow set of test groups of direct co-culture were set up which are; T1: CVMSCs + MDA-MB231 and T2: CVMSCs + HUVECs + MDA-MB231. As described above within the tent groups, different concentrations of CVMSCs and MDA-MB231 were used while the concentration for HUVECs remained constant. The concentration of CVMSCs and MDA-MB231 in all the test groups above was set as 1:1, 1:2, 1:3 and 3:1. Similar condition media was used for both CVMSCs and HUVECs on MDA-MB231. After 14 hour, the ring formation was then stained with CD31 (R&D system, Saudi Arabia) and Calcein AM (Santa Cruz Biotech, Saudi Arabia) before being observed under a fluorescent microscope and the evidence was taken at 2x and 10x magnification. All assays were done in five independent replicates.

#### Staining of Calecien AM and CD31

Cells were first fixed with 4% paraformaldhyde for 60 minute at RT and then washed three times with sterile PBS. Later, the cells were then incubated with primary antibody, anti-human CD31 monoclonal antibody diluted in sterile PBS at 1:100 dilution for 60 minute at RT. The cells were then washed three times prior to the staining of Calcien AM at 1:500 dilution in PBS for 30 minutes at RT. The stained samples were then observed under fluorescent microscope (fluorescence EVOS fl microscopes) and images were taken using AMG software. CD31 was used to determine endothelial markers while Calecien AM was used to determine cell viability.

### Staining of cytokines and chemokines in pre-treated CVMSCs with MDA-MB231

This experiment was designed to test whether pre-seeded MDA-MB231 cells in the bottom chamber (0.4 um membrane insert) can express certain secreted factors to attract CVMSCs in the top chamber of a transwell membrane. As described above different concentrations of MDA-MB231 were seeded on the bottom chamber of a transwell membrane. After 24 hour, CVMSCs were added on the top chamber and incubated for 48 hour. Then, only CVMSCs were collected after trypsinization with TrypLE Express Enzyme (Gibco, #12604013) and stained with different cytokines and chemokines by flow cytometry. The ratios of CVMSCs to MDA-MB231 were 1:1 and 1:3. Then, cells were washed with PBS and re-suspended in FACS buffer (0.5% BSA. 0.5% FBS in 1xPBS). Fixation was done at two steps: 50% methanol and 100% methanol for 20min. Cells then were re-suspended in cold blocking buffer (0.5% BSA and 2% FBS in 1x PBS) for 20 min. After that, designated antibodies were incubated with cells suspended in cold FACS buffer for 35 min. The stained markers were as follow; beta Catenin (R&D # IC13292P), IL-10 (R&D# IC2172P), CXCL9/MIG (R&D # IC392F), IL-12/IL-35 p35 (R&D# IC2191P), CXCL4/PF4 (R&D # IC7952F), and CXCL10/IP10 (R&D# IC226P). Washing step was performed with cold FACS buffer just prior to flow cytometry experiment. Flow cytometry detection was performed through gating 10,000 cells using FACS Canto II machine, BD.

### Statistical analysis

The results are expressed as the mean ± SD of the mean, with at least three independent experiments. Tow-way analysis of variance (ANOVA) for statistical evaluations of proliferation and one-way ANOVA was used for migration values by GraphPad Prism 5.03 software (GraphPad Software, San Diego, CA, USA). Angiogenesis Analyzer macro connected Image J software (Gilles Carpentier, http://image.bio.methods.free.fr/ImageJ/?Angiogenesis-Analyzer-for-ImageJ) was used to test different angiogenic parameters and analyzed by tow-way ANOVA. P<0.05 was considered to indicate a statistical significance.

## Ethics of experimentation

The institutional research board at King Abdullah International Medical Research Centre / King Abdulaziz Medical City, Riyadh, Saudi Arabia approved this study #RC15/136R. All placentae were obtained with informed patient consent in this study.

## Conclusions

This study demonstrates the complexity of the interaction between MSCs and TNBC cells and show that, human CVMSC has the ability to reduce the proliferation, and migration of MDA-MB231 cells in addition inhibit their angiogenic ability.

We can exclude the possibility that the detected decrease or inhibition of proliferation/migration and angiogenesis is caused by exhaustion of the culture medium or crowding. First, the medium in the tested cultures showed a normal color, as in control cultures. Second, Calcein AM staining for viable cells and the observation that the addition of CVMSC led to an even higher inhibition support this conclusion. Third, the addition of control cells (*i*.*e*., untreated cancer cells for proliferation and migration and HUVECs for angiogenesis) did not result in reduction/inhibition of proliferation, migration and angiogenesis.

Quantitative determination of angiogenesis using different angiogenic indexes provided the evidence that CVMSCs reduced/inhibited angiogenesis of TNBC cells in does dependent manner and therefore, these preclinical results suggest that human CVMSCs are potential therapeutic candidate for this aggressive class of BC.

## Supporting information

S1 Fig The positive expression of CD31 marker on the isolated HUVECs using flow cytometry. Gray area is blank and white is CD31.
(TIF)

## Abbreviations

MSCs: Mesenchymal stem cells
ER: Ostrogen receptor
PgR: Progesterone recepto
TNBC: Triple-negative
BC: Breast Cancer
CVMSC: Chorionic villi derived MSCs
HUVECs: Human umbilical vein endothelial cells
TME: Tumour microenvironment
PDGFRβ: Platelet-derived growth factor receptor β’s
ECM: Extracellular matrix
PBS: Phosphate-buffered saline
BSA: Bovine serum albumin
CSCs: Cancer stem cells
FBS: Fetal bovine serum
RT: Room temperature
