# Supplementary figures and images for: The effect of human placental chorionic villi derived mesenchymal stem cell on triple-negative breast cancer hallmarks
Source: PLoS One. 2018 Nov 20;13(11):e0207593. doi: 10.1371/journal.pone.0207593 (PMC6245746; doi:10.1371/journal.pone.0207593)

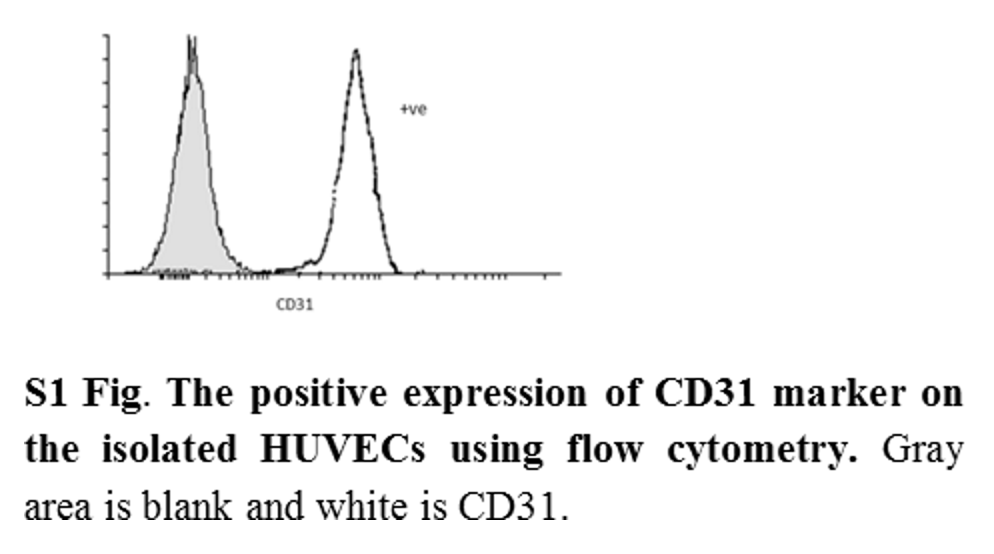

Supplement: S1 Fig — Gray area is blank and white is CD31. (TIF) [file pone.0207593.s001.tif]
